# Selective serotonin reuptake inhibitors: New hope in the fight against COVID-19

**DOI:** 10.3389/fphar.2022.1036093

**Published:** 2022-11-30

**Authors:** Mahsa Asadi Anar, Elaheh Foroughi, Elika Sohrabi, Samira Peiravi, Yasaman Tavakoli, Mozhgan Kameli Khouzani, Parisa Behshood, Melika Shamshiri, Arezoo Faridzadeh, Kimia Keylani, Seyedeh Faride Langari, Akram Ansari, Amirmohammad Khalaji, Setareh Garousi, Mehran Mottahedi, Sara Honari, Niloofar Deravi

**Affiliations:** ^1^ Student Research Committee, School of Medicine, Shahid Beheshti University of Medical Sciences, Tehran, Iran; ^2^ School of Medicine, Isfahan University of Medical Sciences, Isfahan, Iran; ^3^ Department of Medicine, Islamic Azad University of Medical Sciences, Tehran, Iran; ^4^ Department of Emergency Medicine, Mashhad University of Medical Sciences, Mashhad, Iran; ^5^ Department of Medicine, Mazandaran University of Medical Sciences, Sari, Mazandaran, Iran; ^6^ Department of Biology, Science, and Research Branch, Islamic Azad University, Tehran, Iran; ^7^ Department of Microbiology, Young Researchers and Elite Club, Islamic Azad University, Shahrekord, Iran; ^8^ School of Medicine, Kermanshah University of Medical Sciences, Kermanshah, Iran; ^9^ Department of Immunology and Allergy, School of Medicine, Mashhad University of Medical Sciences, Mashhad, Iran; ^10^ Immunology Research Center, Mashhad University of Medical Sciences, Mashhad, Iran; ^11^ School of Pharmacy, Shahid Beheshti University of Medical Sciences, Tehran, Iran; ^12^ Department of Ophthalmology, Labbafinejad Medical Center, Shahid Beheshti University of Medical Sciences, Tehran, Iran; ^13^ Shantou University Medical College, Shantou, Guangdong, China; ^14^ School of Medicine, Tehran University of Medical Sciences, Tehran, Iran; ^15^ Faculty of Medicine, Mashhad University of Medical Sciences, Mashhad, Iran

**Keywords:** antidepressants, COVID-19, SARS-CoV-2, SSRIs drugs, coronavirus, fluoxetine (Prozac), fluvoxamine (Luvox), sertraline (Zoloft)

## Abstract

The emerging COVID-19 pandemic led to a dramatic increase in global mortality and morbidity rates. As in most infections, fatal complications of coronavirus affliction are triggered by an untrammeled host inflammatory response. Cytokine storms created by high levels of interleukin and other cytokines elucidate the pathology of severe COVID-19. In this respect, repurposing drugs that are already available and might exhibit anti-inflammatory effects have received significant attention. With the *in vitro* and clinical investigation of several studies on the effect of antidepressants on COVID-19 prognosis, previous data suggest that selective serotonin reuptake inhibitors (SSRIs) might be the new hope for the early treatment of severely afflicted patients. SSRIs’ low cost and availability make them potentially eligible for COVID-19 repurposing. This review summarizes current achievements and literature about the connection between SSRIs administration and COVID-19 prognosis.

## Introduction

Severe acute respiratory syndrome coronavirus 2 (SARS-CoV-2 virus) can cause COVID-19, an infection with respiratory manifestations ranging from a mild cough and sore throat to severe pulmonary presentation. Common respiratory symptoms include chest pain, dry cough, nasal congestion, shortness of breath, and sore throat (Mouffak et al., 2021). Coronavirus emerged in December 2019 in China (Kaur et al., 2021). Later, the Word Health Organization (WHO) announced a pandemic in March 2020. It is reported that from the beginning of the outbreak until October 2022, 626,337,154 individuals got infected with the virus (COVID-19, 2021).

The lack of effective treatment and its rapid outbreak made researchers look over all accessible medications to find curative therapies for patients suffering from COVID-19 (Hoertel et al., 2021). There are currently multiple vaccines against COVID-19 to reduce mortality and prevent new cases such as weakened or inactivated viruses, engineered RNA or DNA vaccines, viral vector vaccines (non-replicating), and protein-based vaccines (Shiravi et al., 2021). Although a complete cure is not available for all COVID-19 patients now, anti-viral drugs like remdesivir, fluid management, and oxygenation with a ventilator are used for disease management (Gavriatopoulou et al., 2021; Pashaei, 2021). Recent studies suggest selective serotonin reuptake inhibitors (SSRIs) as new hopes to fight against COVID-19 and reduce mortality among COVID-19 patients (Hoertel, 2021; Wroe et al., 2022). Using antidepressants for patients in the intensive care unit (ICU) (2.1% of the total in this study (Hoertel et al., 2021)) reduced the risk of death and intubation. In another multicenter cohort study analyzing health records of 83,584 patients diagnosed with COVID-19, including 3401 patients who were prescribed SSRIs, a reduced relative risk of mortality was found to be associated with the use of SSRIs—specifically fluoxetine—compared with patients who were not prescribed SSRIs (Oskotsky et al., 2021).

Furthermore, some mechanisms are associated with using SSRIs and reducing the death rate (Hoertel et al., 2021). This association was only detected in patients receiving SSRIs when visiting the hospital and not in those who used it only within 3 months before the hospital entrance (Gavriatopoulou et al., 2021). In this review, we comprehensively investigate the effects of SSRIs on the mortality rate and management of ICU patients with COVID-19.

## SSRI

Emerging pandemics such as SARS-CoV-2, which causes COVID-19, have provoked scientists to search for effective treatments. Although antiviral drugs and vaccines are needed, a combination of several drugs and repurposing already available medications can affect different stages of the circulation of COVID-19 and could be the new hope for the treatment of COVID-19.

Before the Corona virus pandemic, several *in vitro* studies had found that functional inhibitors of ASM (FIASMAs) disrupt the flow of infection by intracellular bacterial pathogens, Ebola virus, severe acute respiratory syndrome coronavirus (SARS-CoV) or middle east respiratory syndrome coronavirus (MERS-CoV). Among FIASMAs with antiviral activity, SSRIs have been suggested as sertraline in Ebola virus disease or fluoxetine in influenza virus infection, in hepatitis C virus, coxsackievirus, enterovirus and Ebola virus. In addition to antiviral features, SSRIs have anti-inflammatory and immunomodulary properties that should be under spotlight for the management of inflammatory lung disease in COVID-19. Retrospective observational studies and prospective clinical trials in patient population of Coronavirus have proposed SSRIs as new frame of treatment for the disease management (Zheng et al., 2022).

## Action mechanism of SSRIs

### Anti-inflammatory and immunomodulatory

SSRIs regulate inflammatory cytokine activity and gene expression in the cells (Taler et al., 2007; Hamed and Hagag, 2020; Kuriakose et al., 2021; Kuriakose et al., 2021; Sen, 2021). The key inflammatory pathways such as the signal transducer and activator of transcription 3 (STAT3) and nuclear factor (NF)–ĸB pathways are known to be damped by SSRIs (Lim et al., 2009; Lopez, 2021). SSRIs decreased plasma levels of four of 16 tested inflammatory mediators, including interleukin (IL)-10, tumor necrosis factor (TNF)-α, and CCL-2, which are associated with COVID-19 severity (Lee and Giuliani, 2019; Hojyo et al., 2020), and IL-6, which highly plays a role in disease mortality (Aziz et al., 2020; Hojyo et al., 2020; McElvaney et al., 2020; Tay et al., 2020; Ye et al., 2020).

SSRIs inhibit TNF-α production in inflammation and impair TNF-α release from microglia and monocytes (Lu et al., 2019). TNF-α affects norepinephrine secretion reversibly with SSRIs (Ignatowski et al., 1997). The inflammatory processes involved in the COVID-19 disease, converge with the mechanism in acute and chronic diseases of the lung, and SSRIs modify these routes at several disparate points: pro-inflammatory transcription factors inhibition, depletion of inflammatory cytokines production through canonical serotonergic mechanisms, and suppression of inflammatory cellular responses (Costa et al., 2020).

Depression increases levels of inflammatory cytokines at baseline, and cytokines modulate the hypothalamic-pituitary-adrenocortical (HPA) axis, which ultimately results in increased production of corticotropin-releasing hormone and glucocorticoid receptor resistance (Costa et al., 2020); and diminution of negative feedback at glucocorticoid receptors results in dysregulation of proinflammatory response. Clinical studies revealed the capacity of antidepressants to modulate the function of glucocorticoid receptors in humans (Costa et al., 2020).

Extracellular serotonin concentrations increase by inhibiting serotonin reuptake, which is the action of SSRIs (Tavoulari et al., 2009). SSRIs block the serotonin transporter (SERT), which is expressed in T cells, platelets, and other immune cells (Holinstat and Reviews, 2017). 5-HT (5-hydroxytryptamine) is not just a neurotransmitter but also possesses immunomodulatory properties, inhibiting inflammatory responses by both central and peripheral mechanisms (Costa et al., 2020). The anti-inflammatory properties of SSRIs make them potential candidates for COVID-19 (Kiumarth et al., 2021).

### Carboxypeptidase A3 and serotonin

One study verified that serum levels of carboxypeptidase A3 (CPA3) increased in patients with COVID-19, and interestingly, serotonin levels decreased in serum from SARS-CoV-19 infected patients (Soria-Castro et al., 2021). The primary source of CPA3 in serum is unknown, but CPA3 is expressed in different human organs, such as the lungs (Uhlén et al., 2015). CPA3 is a large protein enzyme in mast cell granules. After cell degranulation of mast cells, which is connected to allergic pathologies of the respiratory tract, CPA3 is released.

Incidentally, an article showed that CPA3 correlated with clinical restrictions associated with systemic inflammation over COVID-19 (Soria-Castro et al., 2021). Mast cell activation is essential to recruit macrophages in infection (Soria-Castro et al., 2021).

Serotonin is also the second important marker that is modified over SARS-CoV-2 infection. (Soria-Castro et al., 2021). Different cells produce blood serotonin, such as mast cells and enterochromaffin of the intestine (Soria-Castro et al., 2021). The preliminary decision of the article has shown that serum levels of serotonin and CPA3 are affected by SARS-CoV-2 infection. Therefore, they can be considered in patients infected with COVID-19 (Soria-Castro et al., 2021).

### Association of anosmia, ageusia, chemesthesis dysfunction, and a changed severity of illness with serotonin deficiency

Anosmia (loss of the sense of smell) and hyposmia/microsomia (reduced perception of smell) are both known as COVID-19 clinical symptoms and represent an early stage of the disease. There are many cases in which olfactory dysfunction is accompanied by gustatory dysfunctions such as ageusia (complete loss of taste) or hypogeusia (reduced taste ability). Several cross-sectional studies have shown that incidence rates vary considerably (based on gender, country, and methodology). Coronavirus patients with a female predominance were more likely to suffer from olfactory (5–95%) and gustatory dysfunctions (38–98%) (Marchese-Ragona et al., 2020). in the study done by Rosario Marchese-Ragona et al. Among the cured patients who had residual gustatory and/or olfactory dysfunction, 53.9% complained of isolated olfactory dysfunction, while 22.5% of isolated gustatory dysfunction and 23.6% of both (Marchese-Ragona et al., 2020).

SARS-CoV-2 patients suffer from depletion of tryptophan, as angiotensin-converting enzyme 2 (ACE2) is a critical element in the absorption of tryptophan from food. This significant patient reduction happens; because coronavirus uses ACE2 as the receptor to enter the host cells. Tryptophan depletion results in a deficit of serotonin (5-HT) in SARS-COV-2 patients, as tryptophan is the precursor in the synthesis of 5-HT. 5-HT is not just a neurotransmitter but also has possess modulatory properties, inhibiting inflammatory responses by both central and peripheral mechanisms (Gordon D. E. et al., 2020). This 5-HT deficiency can explain anosmia, ageusia, and dysfunctional chemesthesis in COVID-19. Since 5-HT is an important neuromodulator in the olfactory neurons, taste receptor cells and transient receptor potential channels (TRP channels) play a role in chemesthesis. In addition, 5-HT deficiency worsens silent hypoxemia and depresses hypoxic pulmonary vasoconstriction and leading to increased disease severity. Also, the levels of anti-inflammatory melatonin (synthesized from 5-HT) and nicotinamide adenine dinucleotide (NAD+, produced from niacin, whose precursor is the tryptophan) might decrease in COVID-19 patients resulting in the aggravation of the disease. Interestingly, SSRIs may not help in correcting the 5-HT deficiency in COVID-19 patients, as their efficacy decreases significantly when there is a depletion of tryptophan in the system (Sen, 2021). The combination of SSRIs and tryptophan should be avoided as a preventive measure to protect against serotonin syndrome. The risk of eosinophilia-myalgia syndrome caused by tryptophan in susceptible patients should also be considered (Sen, 2021).

## Anti-viral

Many SSRI antidepressants, entailing fluoxetine and fluvoxamine, belong to a class of functional inhibitors of acid sphingomyelinase (FIASMA). FIASMA constitutes repeatedly used medications in clinical practice containing non-SSRI antidepressants (e.g., amitriptyline), antihistamines (e.g., hydroxyzine), and calcium channel blockers (e.g., amlodipine), cholesterol drugs (e.g., fenofibrate), and mucolytics (e.g., ambroxol). These medications inhibit acid sphingomyelinase (ASM) both *in vivo* and *in vitro* and catalyze the hydrolysis of sphingomyelin into ceramide and phosphorylcholine (Lu et al., 2019).

Preclinical data suggest that the ASM-ceramide system gets activated by SARS-CoV-2, which leads to forming of ceramide-enriched membrane domains that ease viral access and infection by arraying ACE2 (the cellular receptor of SARS-CoV-2), and the release of pro-inflammatory cytokines. The suppression of the ASM-ceramide system by FIASMA arrests infection of Vero E6 cells with SARS-CoV-2. The disease can be restored by reconstituting ceramides in cells undergoing treatment *via* these antidepressants. The plasma level of ceramide correlates with the severity of clinical manifestation and inflammation markers in COVID-19 patients (Lu et al., 2019).

Sigma-1 receptor (Sig-1R) ligands are frequently identified *in vitro* drug repurposing screens aiming to identify anti-viral compounds against coronaviruses, including SARS-CoV-2. Sig-1R regulates key mechanisms of the adaptive host cell stress response and takes part in the early steps of viral replication. It is enriched in lipid rafts and detergent-resistant endoplasmic reticulum (ER) membranes, where it colocalizes with viral replication proteins. Indeed, the non-structural SARS-CoV-2 protein Nsp6 interacts with Sig-1R (Vela, 2020).

Sig-1R is naturally found at the mitochondria-associated endoplasmic reticulum membranes (MAM); however, when cells experience stress (as in viral infections) Sig-1R translocates to the peripheral ER network and plasma membrane to modulate an array of cell surface proteins (Su et al., 2010). The Sig-1R chaperone is an inter-organelle signaling modulator (Su et al., 2010), which might account for ligand-operated, Sig-1R-mediated modulation of virus attachment or entry. In a study conducted by Gordon et al., Several compounds were found to be inhibitors of SARS-CoV-2 replication *via* action on Sig-1R, suggesting a potential anti-viral feature at the site (Gordon D. E. et al., 2020). The potency of investigated drugs in the study, including fluvoxamine to inhibit replication, was not linked to Sig-1R. However, which pharmacological activity (i.e., agonist or antagonist) of Sig-1R ligands is responsible for the activity of SARS-CoV-2 replication remains uncertain (Hashimoto and neuroscience, 2021). In a later study (Gordon D. E. et al., 2020) they found that knockdown or knockout of SIGMAR1 (encoding Sig-1R) was strongly associated with the dwindling of SARS-CoV-2 replication. It suggests that Sig-1R as functional host-dependency factors for SARS-CoV-2, thus a key target for COVID-19 agent replication (Hashimoto and neuroscience, 2021). With this in hand, fluvoxamine, an effective Sig-1R agonist with immunomodulatory functions in animal studies, has been employed in the trials further to inspect the potential anti-viral properties (Oussama Kacimi et al., 2021). Many theories investigate that inhibition of platelet aggregation, mast cell degranulation, and lysosomotropic associate the mechanisms that fluvoxamine might take to intervene in COVID-19 affliction efficiently (Navin et al., 2020).

The coronavirus disease 2019 pandemic causes short-term and long-term complications in survivors after infection of SARS-CoV-2. The frequent sequelae involve neurologic symptoms (i.e., headaches, memory deficits, difficulty concentrating, cognitive impairment), psychiatric symptoms (i.e., depression, anxiety, sleep disorders), pulmonary abnormalities (i.e., dyspnea, cough, increased oxygen requirement, pulmonary diffusion abnormalities, chest imaging abnormalities), and functional mobility impairment (i.e., impairment in general functioning, mobility decline, reduced exercise tolerance). However, there are no therapeutic drugs for long-term symptoms in survivors of COVID-19 (Hashimoto et al., 2022a).

As evident show, S1R agonists prevent inositol requiring enzyme 1α (IRE1) from splicing of mRNA that encodes X-box binding protein-1 (XBP-1). Hence S1R-mediated reduction in XBP1 activation modulates the ER stress response pathway and reduces cytokine storm (Rosen et al., 2019). plays a central role in the reactivation of the Epstein-Barr virus (EBV). It has been indicated that ER stress and unfolded-protein response induce the expression of the lytic EBV gene in EBV-infected cells, suggesting a pathway in virus-associated complications (Khani and Entezari-Maleki, 2022).

Gold et al. (2021) reported that nearly 70% of cases with long COVID-19 *versus* 10% of the control group were positive for EBV reactivation according to the early antigen-diffuse immunoglobulin G or EBV viral capsid antigen immunoglobulin M.

These patterns suggest that the majority of the long COVID-19 symptoms following the recovery from the disease might not be directly caused by Corona virus but probably result from COVID-19-associated inflammation and EBV reactivation. Considering the link between EBV replication and XBP1 activation and modulatory effects of S1R agonists in XBP1 and ER stress response, it can be proposed that fluvoxamine might have positive effects in abiding symptoms of COVID-19. Nevertheless, further clinical studies are required to confirm this hypothesis (Khani and Entezari-Maleki, 2022).

## Fluvoxamine

High to moderate affinity of SSRIs at the Sig-1R in the rat brain was reported in 1996 by (Narita et al., 1996). The potency at this site has been demonstrated to be the following order: fluvoxamine > sertraline > fluoxetine > escitalopram > citalopram > paroxetine (Narita et al., 1996).

Studies on *in vivo* mice have shown that fluvoxamine improved phencyclidine (PCP)-treated cognitive deficits in mice *via* Sig-1R activation, whereas sertraline and paroxetine did not improve PCP-treated cognitive deficits in the model; these investigations suggest that potential agonism for fluvoxamine and antagonism for sertraline at Sig-1R (Hashimoto et al., 2007).

Improvement of phencyclidine-induced cognitive deficits in mice by subsequent subchronic administration of fluvoxamine, but not sertraline (Hashimoto et al., 2007; Kunitachi et al., 2009).

Fluvoxamine is the most potent Sig-1R agonist among available antidepressants (Hashimoto, 2021). Modulation of the Sig-1R-IRE1 pathway is beneficial in preclinical models of inflammation and sepsis (Rosen et al., 2019). Demonstrated that the Sig-1R is essential for cytokine production in a mouse model of septic shock and that fluvoxamine could protect against inflammatory response and lethal septic shock. Taken together, it is likely that the potent Sig-1R agonists, such as fluvoxamine, might ameliorate inflammatory events (i.e., cytokine storm) associated with ER stress due to SARS-CoV-2 replication (Hashimoto et al., 2022c).

The current therapeutic options for COVID-19, unfortunately, remain expensive and widely unavailable. Fluvoxamine is among the few drugs that have demonstrated therapeutic potential and safety profile in a double-blind, randomized clinical trial (RCT) in humans (Reis et al., 2022). Antidepressant and anxiolytic effects seem to possess anti-inflammatory and immunomodulatory properties (Sen, 2021; Hashimoto et al., 2022b). As a potential lysosomotropic agent, it could also be capable to influences endolysosomal trafficking and prevents the hypercoagulative state of COVID-19 (Taler et al., 2007; Rosen et al., 2019). Fluvoxamine was demonstrated to lower the expression of IL-1, IL-6, and TNF in the striatum of depressed mice (Dallé et al., 2017). A recent RCT indicates that fluvoxamine, an SSRI, sigma-1 receptor (S1R) agonist, and FIASMA may prevent clinical deterioration in outpatients with acute COVID-19 compared to placebo (Lenze et al., 2020). The high affinity of fluvoxamine for the Sig-1R gives it significant immunomodulatory properties (Rosen et al., 2019).

The mechanism by which antidepressants show anti-inflammatory properties is not yet precise. However, there is an information paucity on the role of disturbed and negative emotions on the immune system functionality in recent studies on fluvoxamine (Lu et al., 2019; Hojyo et al., 2020; Ye et al., 2020). Severe clinical manifestations and lack of efficient treatment cause many COVID-19 patients with mental health problems such as depression and post-traumatic stress symptoms (Costa et al., 2020). The HPA axis could be triggered by acute negative emotions, which increase corticotropin levels. This infection leads to immunosuppressive effects (Sperner-Unterweger, 2005). The relation between anti-COVID-19 effects and the moderating role of negative emotions can be tested in RCTs; the results of these studies can demonstrate if antidepressants are advantageous in treating COVID-19 independently of its ameliorating effects on negative emotions or not (Aziz et al., 2020).

SARS-CoV-2 activates ASM and ceramide; which leads to ceramide-enriched membrane domains that present viral entry and infection of the ACE2 group, the cellular receptor for SARS-CoV-2 (Kornhuber et al., 2022). The disease of freshly isolated human nasal epithelial or cultured cells with SARS-CoV-2 was prevented by antidepressants (such as fluoxetine, escitalopram, and sertraline) inhibiting ASM (Carpinteiro et al., 2020). ASM is also known to be inhibited by the fluvoxamine (Kornhuber et al., 2011). Fluvoxamine is suggested to treat compulsions in patients with obsessive-compulsive disorder (OCD). Depending on its formulation, fluvoxamine has a half-life of 9–28 h, and its recommended dose is 100–300 mg/day (Goldenberg, 2012). By reducing serum serotonin by >80% and decreasing the neutrophil recruitment (Schloer et al., 2020), SSRIs can increase bleeding time or reduce the serum serotonin (Härtter et al., 2001).

SARS-CoV-2 infection-induced hyper-inflammation links to the acute lung injury and COVID-19 severity. ACE2 which is central to SARS-CoV-2 entry into cells, is found in mast cells, indicating that they could serve as hosts for this virus (Theoharides 2020). Mast cells (MCs) are strategically located at the mucosa and take role in immune inflammations. The histamine release from mast cells is also decreased by antidepressants (Ferjan and Erjavec, 1996). Protease-1 expression was reduced by SSRIs like fluoxetine in mast cells (Chen et al., 2008). As a result, SSRIs like fluvoxamine may decrease cytokine storms in patients with COVID-19 due to the unusual response of mast cells to SARS-CoV-2 (Chen et al., 2008). Fluoxetine and fluvoxamine act as lysosomotropic Sig-1R agonists (Hallifax and Houston, 2007; Kazmi et al., 2013). β-coronavirus, like SARS-CoV-2, escapes infected cells by using lysosomal trafficking (Ghosh et al., 2020). As β-coronaviruses egress from infected cells through lysosomes, lysosomotropic drugs such as fluvoxamine may have antiviral outcomes in lysosomes containing viruses (Homolak and Kodvanj, 2020). In Vero-E6 cell lines, fluoxetine can inhibit the entry and propagation of SARS-CoV-2 (Schloer et al., 2020). Through inhibition of CYP1A2, an enzyme of the cytochrome P450 family, fluvoxamine can elevate melatonin levels (Härtter et al., 2001).

When comparing the cost of fluvoxamine treatment with the current treatment options, such as remdesivir, bamlanivimab, and casirivimab/imdevimab, a complete 15-day treatment with fluvoxamine would be 70, 37, or 45 times more affordable per patient, respectively (Marčec and Likić, 2021). If it is used early in COVID-19 outpatients, it could prevent many hospitalizations, thus reducing patient mortality, improving the allocation of healthcare resources, and creating significant savings in healthcare costs. Health professionals and decision-makers should become aware of the therapeutic potential of fluvoxamine for COVID-19 patients (Marčec and Likić, 2021). SSRIs have a benign safety profile and are among the most prescribed drugs worldwide. Most side effects of SSRIs (including sexual dysfunction, drowsiness, weight gain, and insomnia) are mild, and most settle within a few weeks of treatment initiation (Costa et al., 2020).

Clinical data suggest that a 10-day course of fluvoxamine probably reduces the risk of death and intubation among early-stage COVID-19 patients. For mild to moderate depression, fluvoxamine is the most appealing antidepressant (Hashimoto et al., 2022b; Marzolini et al., 2022).

Lenze et al. (2020), decided to test fluvoxamine to treat COVID-19. They performed a double-blind RCT on 115 outpatients screened for COVID-19. Immediately after diagnosis, the intervention was started with 50 mg of fluvoxamine, then 100 mg twice daily for 2 days; after tolerance, 100 mg 3 times daily for 15 days, and the same amount was given. Another similar group was given placebo. Patients in both groups were followed for shortness of breath and decreased oxygen saturation to less than 92% and hospitalization for these reasons and due to pneumonia within 15 days and then 1 month after infection. Although the sample size was small, none of the patients treated with fluvoxamine met the criteria for shortness of breath or hospitalization due to pneumonia, compared with six patients in the placebo group.

At the same time, Dr. Nicolas Hoertel in France observed that despite the virus spreading in all sections of French society, the incidence of COVID-19 in psychiatric patients was lower. He and colleagues evaluated a database of 15,000 patients at 39 affiliated hospitals. They concluded that antidepressants “could be associated with lower risk of death or intubation in hospitalized patients with COVID-19.” (Hoertel et al., 2021).

On the other side, when the outbreak of COVID-19 on the racetrack of Berkeley happened, Dr. David Seftel, chief executive officer of Enable Biosciences and a veteran physician at Berkeley Golden Gate Fields, learned the results of the two studies about fluvoxamine and decided to suggest it to volunteer staff. None of the 77 employees at Golden Gate Fields who opted to take fluvoxamine required hospitalization, compared with a 12.5% hospitalization rate for the 48 employees who declined the drug (Lopez, 2021).

Fluvoxamine therapy for 10 days decreases tertiary hospitalization by one-third compared with a placebo. It also increases heme oxygenase (HO)-1, which is blocked in COVID-19 infection. HO-1 has anti-inflammatory effects and reduces tissue damage caused by reactive oxygen species (ROS) (Hooper, 2022).

The fluvoxamine treatment’s effectivity on membrane trafficking was dependent on the cell line. Though a standard dose of fluvoxamine causes noticeable brain enrichment, its convergence in the blood plasma is low. Treatment for 1 hour caused a meaningful increase in the endocytosis (Kasper et al., 1993). They showed that sub-therapeutic doses of fluvoxamine may rapidly change endocytosis in a non-specific way. It shows that it can also affect the endocytic trafficking of the SARS-CoV-2 (Glebov, 2020). To inspect its possibility, the essential steps of SARS-CoV-2 endocytosis are recreated by imaging internalizing the recombinant SARS-CoV-2 spike protein to HEK293T cells transfected with a plasmid signifying the human SARS-CoV-2 receptor ACE2 (McKay et al., 2020).

In HEK293T cells, the effect of fluvoxamine with a range of fluvoxamine concentrations on membrane trafficking of SARS-CoV-2 spike proteins and their cell host receptor, ACE2, was investigated. A sub-therapeutic concentration (80 nM) of fluvoxamine, increased fluid-phase endocytosis, and an influx of spike-ACE2 molecules into larger early endosomes (Glebov, 2021).

Fluvoxamine with a dose of 80 nM in 1 h resulted in a significant increase in spike protein, but relative levels of spike protein surface-bound were not changed within 1 h. According to these results, it can be concluded that fluvoxamine treatment can upregulate the endocytosis of spike protein (Glebov, 2021). At high concentrations, various antipsychotics and antidepressants may affect lipid dynamics by inhibiting direct manipulation of the lipid bilayer or ASM (Kapoor et al., 2019).

The RCT study was done on the effect of fluvoxamine on 152 outpatients with symptomatic COVID-19. Participants were randomly assigned to receive 100 mg of Fluvoxamine (*n* = 80) or a placebo (*n* = 72) 3 times daily for 15 days. Fluvoxamine was correlated with a decrease in clinical regression in adults with COVID-19 (Lenze et al., 2020).

Rudez et al. reported a 46-year-old man with COVID-19. On day nine, after the onset of symptoms, he was administered fluvoxamine 50 mg BID for 14 days. Within 24 h, his olfactory and taste sensations Rebounded, the cough and dyspnea disappeared, and his fever status was resolved. No symptoms remained at the follow-up session after 4 weeks. If indicated early in coronavirus-afflicted outpatients, it seems that fluvoxamine can significantly improve patients’ condition, preventing most hospitalizations and decreasing mortality rates (Rudez et al., 2021).

Seftel and Boulware (2021) examined 65 COVID-19 patients receiving fluvoxamine 50 mg twice daily, and 48 refused. The risk of hospitalization was 0% with fluvoxamine and 12.5% with observation alone. In 14 days, only a tiny percentage of people with fluvoxamine had residual symptoms, while most people who did not take fluvoxamine had residual symptoms.

A prior meta-analysis showed that the application of the antidepressant in major depressive disorder would reduce plasma levels of several pro-inflammatory mediators, which have been associated with severe COVID-19. Recent studies also suggest that several antidepressants may inhibit acid ASM activity, which may prevent the infection of epithelial cells with SARS-CoV-2. Also, the fluoxetine may exert *in vitro* anti-viral effects on SARS-CoV-2 (Hoertel et al., 2021).

Despite the benefits mentioned above, it is essential to note that fluvoxamine has a high potential for drug interactions, which should be considered when administered to patients with COVID-19 (Back et al., 2021).

One study has found increased mortality and morbidity among elders who have taken SSRI (Vozoris et al., 2018). Fluvoxamine is metabolized by cytochrome P450 (CYP), so it can affect other drugs that inhibit this enzyme, which occurs immediately after taking fluvoxamine; however, the treatment period with fluvoxamine is short relatively (10 days) (Reis et al., 2022). On the other hand, fluvoxamine strongly inhibits CYP1A2 and CYP2C19 and inhibits CYP2C9, CYP2D6, and CYP3A4 with moderate potency; therefore, it can increase the effect of drugs that are metabolized by these enzymes (van Harten, 1995).

Despite the beneficial effects, drug interactions can’t be ignored, especially for some drugs (such as antidepressants and antiepileptic drugs), which their doses should be set according to their clinical effects; furthermore, they have a narrow therapeutic range.

Another issue with fluvoxamine is the risk of serotonin toxicity; so using fluvoxamine with monoamine oxidase inhibitors such as phenelzine, linezolid, and tranylcypromine is contraindicated (Back et al., 2021). Another concern about fluvoxamine is prolonging the QT interval when used with other medications with similar risk. Another precaution about fluvoxamine in diabetics is that it may change blood sugar control. It is potentially necessary to adjust the dose of anti-diabetic drugs.

One crucial change is pharmacogenomic differences in CYP2D6 that control metabolic abasement of fluvoxamine. CYP2D6 demonstrates the increased number of functional copies contributing to ultrarapid metabolic actions (Marzolini et al., 2022). It has been discovered that it can be as high as 8.5%. Thus, it is likely that around 63 patients out of the 741 patients who took fluvoxamine in this study, might have had more than two practical copies of the gene, which could increase the probability of their being ultrarapid metabolizers (Marzolini et al., 2022). According to this, their result might be closer to the placebo group. Because genetic variants in CYP2D6 might only be to the point of the active drug cohort, the net effect of not controlling for this variable would be to decrease the observed specific role of the fluvoxamine (Marzolini et al., 2022).

**FIGURE 1:**
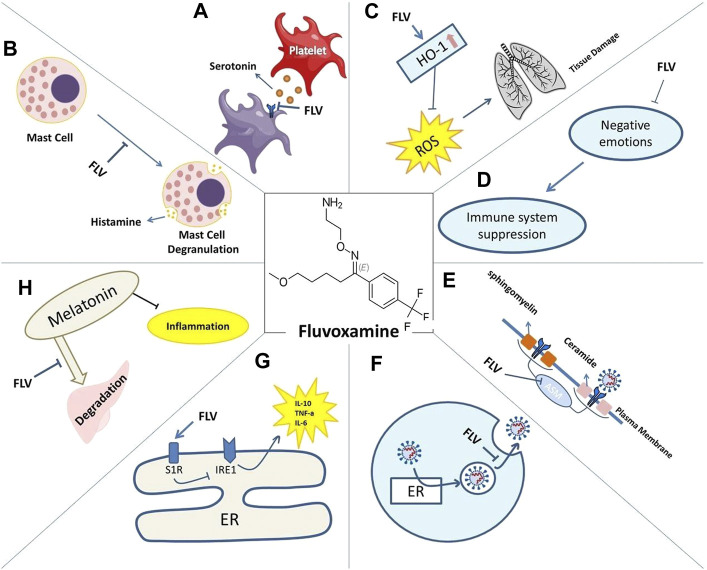
Mechanisms of action of Fluvoxamine for COVID-19 include: reduced serotonin uptake by platelets **(A)**. Reduced histamine release from mast cells **(B)**. Prevented lung tissue damage due to reduced levels of ROS caused by HO-1 increase **(C)**. Restored immune system activity as negative emotions are lessened **(D)**. Impaired plasma membrane binding of ASMase **(E)**. Lysosomotrophic properties interfering with viral trafficking **(F)**. S1R increased activity and its effect on IRE1-induced inflammation **(G)**. Decreased melatonin degradation and its effect on inflammation **(H)**.

There are hypotheses about fluvoxamine that may show the mechanism of its action in COVID-19 and sepsis, including anti-inflammatory effects (reduction of cytokines) induced by Sig-1R activation and modulation of the impact of IRE1 on autophagy and inhibition of platelet activation by SSRIs and direct antiviral effects Through lysosomotropic properties (Schlienger and Meier, 2003; Fung and Liu, 2019; Homolak and Kodvanj, 2020). despite all these hypotheses, the safety, and efficacy of fluvoxamine treatment in COVID-19 should be more carefully tested and validated in other studies with adequate samples. Because many of the drugs that were considered sufficient, when tested carefully enough in clinical trials, are sometimes diagnosed as ineffective or even harmful due to their drug interactions or side effects, etc. (Figure 1).

A recent study published in 2022 (Bramante et al., 2022) investigated the effects of different dosages of fluvoxamine on patient outcomes. It was shown that fluvoxamine at a low dose of 50 mg twice a day prevented a primary event in the ICU patient. In two randomized, double-blind, placebo-controlled trials, researchers found that higher-dose fluvoxamine (100 mg two to three times a day) led to a 25–30% reduction in hospitalization or a prolonged emergency department stay (Reis et al., 2021).

Because agonism of the Sig-1R may be an important mechanism of fluvoxamine against COVID-19, the dose might need to be higher to produce an effect, especially in patients who are overweight or obese.

Apart from methodological stipulation, we think that the widespread use of fluvoxamine is currently inappropriate, and that other intrinsic pharmacological attributes of the drug should be scrutinized that have been mentioned by several authors (Glebov, 2021). Although the adverse events during the clinical trials (Lenze et al., 2020; Reis et al., 2021)on fluvoxamine have been moderate, the incidence of clinically significant drug-drug interactions or of psychiatric adverse reactions of SSRI (e.g., insomnia, anxiety,...) could be a concern on a large-scale use (Blaess et al., 2021). Moreover, fluvoxamine is a not a drug with smart pharmacokinetics. Fluvoxamine has a high first-pass metabolism and displays a non-linear and sex-dependent pharmacokinetics with a high inter-patient variability (Altamura et al., 2015). Fluvoxamine is metabolized by a CYP2D6 that displays a marked racial/ethnic difference in frequency of functional alleles. Therefore more cutting edge evidence is required for prospective large scale use.

## Fluoxetine

Fluoxetine substantially reduces the risk of intubation or death, independently of patient characteristics, clinical and biological markers of disease severity, and other psychotropic medications. This role remained significant in multiple sensitivity analyses. Exploratory analyses demonstrate that SSRI and non-SSRI antidepressants reduce the risk of intubation or death (Hoertel et al., 2021).

SSRIs may benefit patients with COVID-19 owing to the link between serotonin and the immune system (Ahern, 2011; Hamed and Hagag, 2020). More specifically, severe outcomes of COVID-19 are the result of several pro-inflammatory cytokines, including IL-6, whose increased levels contribute to the cytokine storm (Costela-Ruiz et al., 2020). Various studies have indicated that SSRIs, specifical fluoxetine, can decrease levels of these cytokines and IL-6 signaling activity (Służewska et al., 1995; Köhler et al., 2018).

Fluoxetine has an antiviral effect on COVID-19 patients partly because they are associated with decreased plasma levels of pro-inflammatory cytokines *via* the Sig-1R agonism (Fred et al., 2021). The administration of 100 mg of fluvoxamine over 15 days to adult outpatients with COVID-19 reduced the likelihood of clinical deterioration (Lenze et al., 2020). It was also observed that fluoxetine decreased the SARS-CoV-2 titer in men with COVID-19 after a minimum treatment period of 10 days and a dose of at least 20 mg/day (Eugene, 2022). An average dose of fluoxetine of 20 mg/day resulted in a lower risk of death and intubation among a population consisting of 63% females and 37% males (Hoertel et al., 2020; Hoertel et al., 2021). These observational studies also proved fluoxetine’s therapeutic benefit in COVID-19 patients.

Clinical data reveal that many SSRIs decrease IL6 signaling activity and reduce hyperinflammatory states (Basterzi et al., 2005; Hashioka et al., 2007; Taraz et al., 2013; Gobin et al., 2014; Durairaj et al., 2015; Wang et al., 2019). Using fluoxetine to disrupt this NF-_k_B (nuclear factor -_k_B)/IL6ST axis and mitigate the after-effect cytokine storm may increase survival and lower hospitalization rates in COVID-19 patients (Creeden et al., 2021). Also, fluoxetine can improve asthma outcomes and defend against chronic methamphetamine such as pulmonary inflammation (Brown et al., 2018).

As mentioned before, another possible explanation could be the inhibiting result on the ASM/ceramide system (Hoertel et al., 2021), its activation may play a significant role in SARS-CoV-2 infection since it leads to the creation of ceramide-enriched membrane domains that help infection by clustering ACE (Carpinteiro et al., 2020; Carpinteiro et al., 2021). Metabolic markers from the metabolism of ceramide have also been belonging to inflammation in COVID-19 patients who were hospitalized (Marín-Corral et al., 2021). Finally, some proof suggests that SSRIs, especially fluoxetine, can have antiviral effects (Kristiansen and Hansen, 2000; Zuo et al., 2012; Dechaumes et al., 2021).

Entering the host cell is one of the steps in the life cycle of viruses such as SARS-CoV-2. The host cell membrane must be overcome to transfer the genome into the cytosol. This step happens through cellular and viral membranes (Schloer et al., 2020). The antiviral action of fluoxetine is based on the inhibitory effect on ASM. ASM’s block leads to sphingomyelin accumulation, which can approve antiviral barrier against varus (Kornhuber et al., 2010).

Antiviral drugs such as remdesivir or GS-441524 can directly target viral patients and eliminate the pathogen, and they also reduce the risk of viral resistance (Brunotte et al., 2021; Schloer et al., 2021).

On the other hand, combination therapies that include host-directed drugs and viruses can cause less defiance. It reported that the balance of endosomal lipids is essential for the entry process of viruses like SARS-CoV-2. The clinically certified antidepressant fluoxetine converts ASM within the lysosomal/endosomal separations (Kornhuber et al., 2010). Fluoxetine’s inhibitory influences depend on its capability to mediate the lysosomal lipid balance to prevent the entry of SARS-CoV-2 (Schloer et al., 2020).

Yan VC et al. reported that the ribonucleic acid (RNA) polymerase inhibitor, remdesivir, combined with used fluoxetine, expresses a synergistic antiviral effect in contract with the SARS-CoV-2 infection *in vitro*. Remdesivir easily converts into its primary plasma metabolites (Yan and Muller, 2020).

Brunotte L et al. showed that the combination of GS-441524 and fluoxetine can lower the viral titers synergistically compared to monotherapy. Whereas monotherapy could reduce viral titers by around 60%–70%, in combination therapy above 90%, this drug combination is safe, and there was no toxicity (Brunotte et al., 2021).

Schloer S et al. found that fluoxetine, as a FIASMA, efficiently inhibited the entry and spread of SARS-CoV-2 in the cell culture model and also exerted potent antiviral activity. Enveloped viruses usually use the host cell endocytic gadgets for a safe transit into the cell (Schloer et al., 2020). A critical move in the infection cycle is the combination of the virus envelope with the cell membrane to move the viral genome into the cytosol. Although the influenza A virus transfers the viral genome by fusion with the endosomal membrane, SARS-CoV-2 can merge straight with the plasma membrane. In contrast, new evidence confirms that SARS-CoV-2 also uses endocytic uptake to enter the host cell. A block of SARS-CoV-2 entry was only reached when the proteolytic activities of TMPRSS2 at the plasma membrane and the endolysosomal cathepsins were blocked (Schloer et al., 2020). Previous results on influenza A virus infection showed that rising endolysosomal cholesterol levels and the accompanying alterations in luminal pH also impair the SARS-CoV-2 condition. Raised cholesterol levels in the endolysosomal membranes render the vacuolar-type membrane ATPase (Kühnl et al., 2018).

It was reported that fluoxetine inhibited the production of ASM and prevented infection of various cells, including cultured cells and human nasal epithelial cells infected with SARS-CoV-2 and pseudo viral particles containing the SARS-CoV-2 spike protein (Carpinteiro et al., 2020; Kiumarth et al., 2021).

Dechaumes et al. showed that adding 10 μM fluoxetine to Vero E6 cells infected with SARS-CoV-2 could inhibit virus-induced cytopathic *in vitro* compared to the control group (Dechaumes et al., 2021).

As previously observed, fluoxetine obviously obstructs the copying of RNA viruses, especially Coxsackieviruses B, such as CV-B4 *in vivo* and *in vitro* (Alidjinou et al., 2015; Benkahla et al., 2018; Dechaumes et al., 2021).

SARS-CoV-2 is an enveloped RNA virus that comes into the cell *via* pH-dependent endocytosis. The incoming RNA is translated into viral proteins (Zimniak et al., 2021). Antiviral therapies have been developed to inhibit viral polymerase, integrases, and entry. Remdesivir (Goldman et al., 2020), lopinavir (Cao et al., 2020), and chloroquine (Ferner and Aronson, 2020) were introduced to suppress viral replication and had little effect on virus replication *in vitro*, but in human patients lead to severe adverse side effects (Cao et al., 2020; Ferner and Aronson, 2020; Goldman et al., 2020; Spinner et al., 2020). Ribavirin and interferon (Hung et al., 2020) had no significant impact on patient survival rates. Now, monoclonal antibodies such as imdevimab, bamlanivimab, and basiliximab are observed to treat mild COVID-19; But a strong direct antiviral drug is still not accepted.

Zimniak et al. (2021) observed that fluoxetine at a concentration of 0.8 μg/ml diminished viral infection in human lung slices, demonstrating its activity in relevant human tissue targeted in severe COVID-19. Also, it reduces the expression of viral proteins. They found that both isomers of fluoxetine (R-fluoxetine and S-fluoxetine) do the same act on the virus. The antiviral result is irrelevant to the serotonin reuptake receptor since none of the serotonin reuptake transporters like Paroxetine or Escitalopram impede viral copying. Fluoxetine treatment declines protein declaration, showing that fluoxetine is upstream of the gene declaration. Also, by reducing the generation of both RNA and protein, Zuo et al. (2012) Demonstrated the effectiveness of fluoxetine in inhibiting coxsackievirus infection.

Schloer et al. reported that fluoxetine notably abates SARS- CoV-2 titers, after an incubation period, in both human-lung Calu-3 cells (EC50 = 0.82 μM and EC90 = 4.02 μM, MOI = 0.1) and African green monkey kidney epithelial Vero E6 cells (EC50 = 0.69 μM and 90% maximal effective concentration (EC90 = 1.81 μM and MOI = 0.01) (Schloer et al., 2020). Similarly, Hoertel et al. showed in an observational cohort study that COVID-19 patients cured at an average fluoxetine dose of 20 mg had a lower risk of intubation and death (hazard ratio [HR] = 0.32; 95% confidence interval [95% CI] = 0.14–0.73, *p* = 0.007) (Hoertel et al., 2021).

Fred et al. (2021) tested the effect of psychoactive drugs to decrease SARS-CoV-2 infection of host cells *in vitro*. They showed that pharmacologically different antidepressant drugs and several antipsychotics could reduce the disease by pseudo-typed viruses hiding SARS-CoV-2’s protein. Healing human lung epithelial cell line Calu-l affected with the B.1 lineage of SARS-CoV-2 with these drugs also successfully decreased the infectious virus. In addition, infection by pseudotyped viruses, carrying N501Y, K417N, or E484K single point mutations or triple mutant (N501Y/K417N/E484K) in the spike protein was shown to be decreased by fluoxetine. Fluoxetine was also productive against the variants of SARS-CoV-2, VoC-1 (B.1.1.7), and VoC-2 (B.1.351) in Calu-1 cells.

Following the third coronavirus wave outbreak in April 2021 in Hungary (World Health Organization, 2020) and increasing deaths, anxiety increased among patients and the treatment team (Pérez-Cano et al., 2020; Çağlar and Kaçer, 2022). This condition has encouraged clinical therapists to use anti-depressant and sedatives for patients. Although benzodiazepines are the first line of treatment in emergency departments, they cause respiratory depression at high doses and increase the risk of mortality in community-acquired pneumonia (Ely et al., 2012; Sanders, 2013). SSRIs are other well-known drugs for reducing anxiety (Jakubovski et al., 2019). Although the therapeutic effects of SSRIs start with a delay, according to some studies, they can be efficient at the end of the first week (Taylor et al., 2006).

Németh et al. (2021) in Hungary evaluated the medical records of adult patients with moderate to severe pneumonia admitted to the hospital. In the study, patients received a variety of single-drug or combination therapies, including antivirals such as Remdesivir or Favipiravir and Barsitinib, and 110 patients took fluoxetine at a dose of 20 mg once daily as adjunctive therapy. Fluoxetine use was associated with an important (70%) decrease of mortality (OR [95% CI] 0.33 [0.16–0.68], *p* = 0.002) compared to the non-fluoxetine group. This study showed no increased risk of bleeding following fluoxetine use compared to other studies (Laporte et al., 2017; Samuel and Seifert, 2017). Hyponatremia has been reported in only one patient taking fluoxetine, after which the drug was discontinued. Exacerbation of anxiety following the onset of fluoxetine has not been reported, although no detailed test has been performed to measure anxiety during treatment. In this study, Fluoxetine was generally well tolerated by patients with COVID-19, probably due to its minimal antidepressant dosage (20 mg); in comparison, if we use higher doses, the maximum dose (80 mg) may be even more effective against COVID-19 (Németh et al., 2021).

On the other hand, if a combination of drugs with different mechanisms of action is used, therapeutic effects can be achieved with low doses of medications. For example, the virus may be more effectively controlled to fight viral diseases by using a combination of drugs that directly affect the virus at the same time as drugs that affect the host’s cells. In addition, the risk of drug resistance is reduced (Ianevski et al., 2020; Zumla et al., 2020).

In the study in 2021 by Schloer et al. (2021), the effect of concomitant use of Remdesivir, as a drug that acts directly on RNA polymerase virus (Gordon C. J. et al., 2020), simultaneously with an effective medication on the endosomal host-virus interface, such as fluoxetine (Kornhuber et al., 2010; Schloer et al., 2020) which negatively affects endosomal cholesterol release and Itraconazole (Trinh et al., 2017; Schloer et al., 2019), which inhibit the endosomal cholesterol transporter, were assessed. This study found that using a combination of drugs in this *in vitro* environment has effectively controlled the SARS-CoV-2 infection. Similarly, in 2022, Pashaei (2022) hypothesized that using Fluoxetine with Molnupiravir could yield more effective outcomes through its immunomodulatory, anti-inflammatory, and antiviral effects.

**FIGURE 2:**
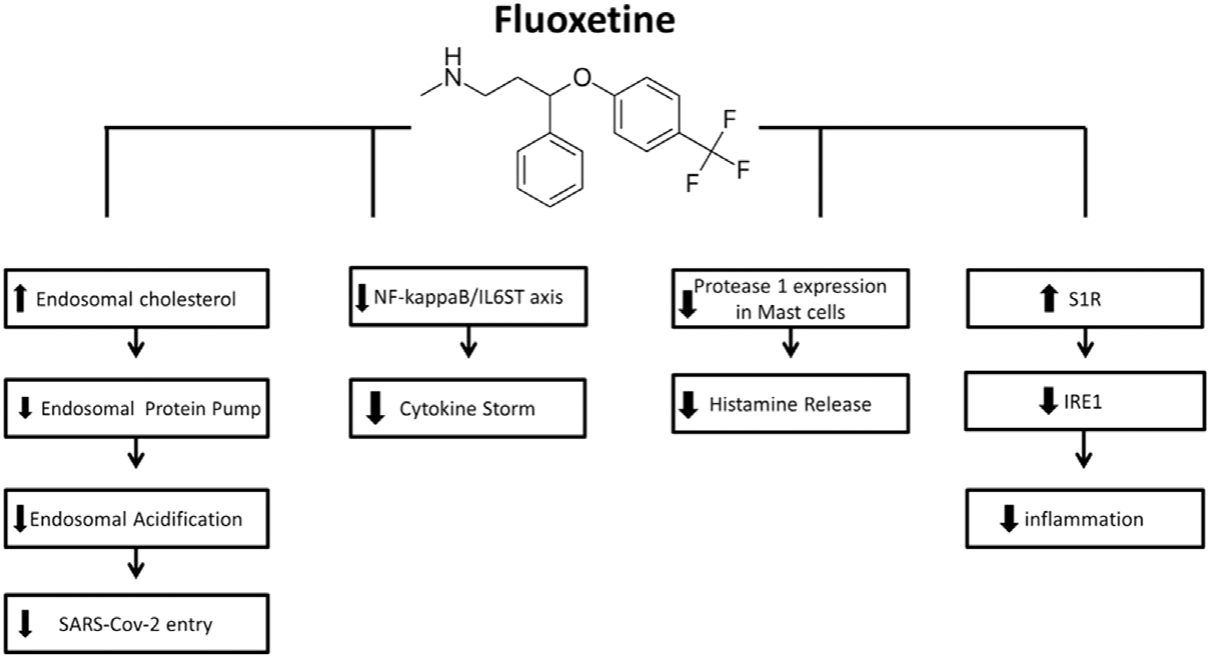
Mechanisms of action of Fluoxetine for COVID-19 (from left to right): FLX can inhibit SARS-CoV-2 entry into cells through the endocytic pathway by inducing accumulation of cholesterol within the endosomes, leading to inactivation of the endolysosomal proton pump manage pH maintenance and so leading to impairment of endolysosomal acidification. The inhibitory effect of FLX on ASMase activity results in sphingomyelin accumulation, providing a barrier against virus entry. FLX suppress the cytokine storm by interfering with NF-kappaB/IL6ST axis. FLX is also found to decrease the protease-1 expression on mast cells and histamine release, hence the decreased cytokine storm. the S1R activation induced by FLX mediates the inhibition of IRE1 and related inflammatory response.

The important point in using a combination of these drugs in the clinical environment is to pay attention to their bioavailability and drug interactions. Fluoxetine interacts with drugs by inhibiting CYP (Spina et al., 2012; Eugene, 2019). According to Bolo et al., fluoxetine concentrations in the brain are 10-fold higher than in human plasma in a steady-state (Bolo et al., 2000). For each tissue, fluoxetine distribution coefficients were as follows: 60 for the lung, 15 for the brain, 10 for the heart, 38 for the liver, 20 for the spleen, and nine higher for the kidneys (Johnson et al., 2007). Therefore, contraindications for use and risk assessment for each patient should be considered separately, according to their specific conditions. (Figure 2)

## Sertraline and paroxetine

COVID-19 showed an increase in pro-inflammatory cytokines (Conti et al., 2020). These raised levels showed an increasing serotonin metabolism by activating indoleamine-2,3-dioxygenase enzyme. This enzyme metabolizes tryptophan which is the precursor of serotonin (Myint and Kim, 2003). Other studies reported that the raised C-reactive protein levels showed depression-like symptoms (Frommberger et al., 1997; Monje et al., 2011).

SSRIs may help COVID-19 patients to prevent cytokine release syndrome responsible for complicating illness progression and increasing TNFα (Roumestan et al., 2007).

STAT3 has a crucial role in the inflammatory loop, and medications that suppress this pathway need attention. Sertraline and paroxetine attenuate mitogen-stimulated increases of STAT3 and Cyclooxygenases-2, which are also involved in COVID-19 immunopathology. This function is more prominent than dexamethasone, another keystone in COVID-19 therapy (Oussama Kacimi et al., 2021).

Paroxetine is a novel COVID-19 therapeutic candidate (Zhou et al., 2020); a study corresponded with antidepressant therapy with declined risk of intubation or death for hospitalized patients with COVID-19 (Hoertel et al., 2021).

Endoplasmic protein Sig-1R are important in the pathophysiology of psychiatric disorders as in the activation mechanisms of some SSRIs. Some SSRIs possess affinity to Sig-1Rs; in descending order: fluvoxamine (Ki = 17.0 nM) > sertraline (Ki = 31.6 nM) > fluoxetine (Ki = 191.2 nM) > citalopram (Ki = 403.8 nM) ≫ paroxetine (Ki = 2041 nM) (Hashimoto, 2009; Ishima et al., 2014). Fluvoxamine, but not paroxetine has a significant affinity for binding to Sig-1Rs in the human brain, and previous articles suggested that the Sig-1R may be involved in action mechanism of some antidepressant drugs specially fluvoxamine (Hashimoto, 2009; Kishimoto et al., 2010). Recent articles have reported that sertraline may be a Sig-1R antagonist, whereas fluvoxamine can be the Sig-1R agonist (Hashimoto, 2009; Hayashi and Stahl, 2009; Kishimoto et al., 2010; Ishima et al., 2014). Fluvoxamine and sertraline are potent for the Sig-1Rs (Ishima et al., 2009), while paroxetine is weak and with no affinity (Hashimoto et al., 2007).

Table 1 summarizes the studies on the effect of SSRIs on the prognosis of COVID-19.

**TABLE 1:** A summary of the studies on the effects of SSRIs on the prognosis of COVID-19.

Author/year	Country	Type of study	Follow up duration	Population	Sex (female)	SSRI	Outcome
Lenze et al. (2020)	Eastern Missouri and southern Illinois (United States)	RCT	15 days	152 patients with COVID-19	72% (70% with fluvoxamine and 53% with placebo)	100 mg of fluvoxamine (*n* = 80) or placebo (*n* = 72) 3 times daily for 15 days	• Clinical deterioration occurred in 0 of 80 patients in the fluvoxamine group and in 6 of 72 (8.3%) patients in the placebo group
							• No fluvoxamine-treated patients met the criteria for clinical deterioration as defined in the study, whereas 8.3% of patients taking a placebo met this endpoint
							• The primary endpoint included shortness of breath or hospitalization for shortness of breath or pneumonia and oxygen saturation dropped below 92% or supplemental oxygen was required to keep oxygen saturation at or above 92%. The prespecified primary outcome analysis was determined instead by survival analysis (time to clinical worsening). The absolute difference and 95% CI are for the Kaplan-Meier estimate of the placebo group at day 15. The test of difference is the log-rank statistic (χ2 = 6.8)) with a *p*-value under 0.05 (0.009) had a significant association with the SSRI-taken group
							• Secondary endpoints like (shortness of breath and oxygen saturation <92% but no supplemental oxygen needed) with a *p*-value of more than 0.05 (0.15) had no significant association with the SSRI-taken group
							• Secondary endpoints like oxygen saturation <92% plus supplemental oxygen needed and hospitalization related to dyspnea or hypoxia with a *p*-value of more than 0.05 (0.07) had no significant association with the SSRI-taken group
							• Secondary endpoints like oxygen saturation <92% plus supplemental oxygen needed and hospitalization related to dyspnea or hypoxia plus ventilator support needed for≥3 days with a *p*-value of more than 0.05 (0.36) had no significant association with the SSRI-taken group
Hoertel et al. (2021)	France	Cohort	January 24th, until April 1st	7230 Adults hospitalized for COVID-19, 345 patients	3555 (49.2%)	Citalopram (N = 21)	• A significant association between antidepressant use and reduced risk of intubation or death (HR, 0.56; 95% CI, 0.43–0.73, *p* < 0.001)
						Escitalopram (N = 63)	• Antidepressant use could be associated with lower risk of death or intubation in patients hospitalized for COVID-19
						Fluoxetine (N = 30)	
						Fluvoxamine (N = 1)	
						Paroxetine (N = 63)	
						Sertraline (N = 22)	
						Vortioxetine (N = 2)	
Seftel and Boulware (2021)	United States	Cohort	7 and 14 days	113 COVID-19 patients	28 (24.8%)	Fluvoxamine 50 mg BD for 14 days (N = 65)	• Higher incidence of subsequent hospitalization in no therapy group (12.5% vs. 0%; *p* = 0.005)
						No therapy (N = 48)	• Respiratory rates improved faster by day 7 with the fluvoxamine (*p* = 0.001)
							• Persistence of ongoing symptoms were higher in control group (60% vs. 0%; *p* < 0.001)
							• No serious adverse events
							• No events leading to discontinuation
Reis et al. (2022)	Brazil	RCT	198 days	9803 patients with COVID-19	58%	fluvoxamine (100 mg twice daily for 10 days)	• The proportion of patients observed in a COVID-19 emergency setting for more than 6 h or transferred to a tertiary hospital due to COVID-19 was lower for the fluvoxamine group compared with placebo (79 [11%] of 741 vs. 119 [16%] of 756)

Bramante et al. (2022)	United States	RCT	May 2021 to February 2022	1431 COVID-19 patients	56%	Fluvoxamine 50 mg BD for 14 days	• Fluvoxamine not prevented the occurrence of followings: COVID-19 associated death, hospitalization, emergency department visit, and hypoxemia (adjusted OR [95% CI] = 0.94 [0.66–1.36]; *p* = 0.75)
Német et al. (2021)	Hungary	Observational retrospective study	17 March and 22 April 2021	623 adults’ patients with moderate to severe pneumonia admitted to the hospital	45.4%	Fluoxetine 20 mg once daily as adjunctive therapy to antiviral drugs	• An important (70%) decrease of mortality (OR [95% CI] 0.33 [0.16–0.68], *p* = 0.002) in patient who receiving fluoxetine adjunctive therapy
							• The survival of patients in the fluoxetine group increased threefold
							• No exacerbation of anxiety in these patients
							• No increased risk of bleeding
							• Well tolerated by patient in this minimal dose
							• No significant hyponatremia (just reported in one patient)
Eugene (2022)			10-day	1000 COVID-19 patients		fluoxetine antidepressant doses (20 mg/day, 30 mg/day, 40 mg/day, 50 mg/day, and 60 mg/day)	• By day-10 at 20 mg/day 93.2% and 47% of the population willachieve the trough target plasma EC50 and EC90 concentrations, respectively, which translates to a lung tissue distribution coefficient of 60-times higher EC50 (283.6 ng/ml [0.82 mM]) and EC90 (1390.1 ng/ml [4.02 mM])
							• Further, by day-10 at an ideal dose of 40 mg/day, 99% and 93% of patients will reach the trough EC50 and EC90 concentrations, respectfully
							• Lastly, only a dose of 60 mg/day will reach the SARS-CoV-2 EC90 inhibitory concentration in the brain
Hoertel et al. (2020)	France	Cohort study	18.5 days	7,345 adults hospitalized with COVID-19	49.3%	Escitalopram (N = 84)	• Unadjusted hazard ratio estimates of the association betweenantidepressant use and the primary outcome stratified by age (i.e., 18-50, 51-70, 71-80, and81+) were non-significant (all *p* > 0.072), except in the group of patients aged 71–80 years (HR, 0.66; 95% CI, 0.45 to 0.98; *p* = 0.041)
						Paroxetine (N = 79)	• There is association between use of any antidepressant (HR, 0.64; 95% CI, 0.51to 0.80; *p* < 0.001), SSRI (HR, 0.56; 95% CI, 0.42 to 0.75; *p* < 0.001), and SNRI (HR, 0.57; 95% CI, 0.34 to 0.96; *p* = 0.034), and reduced risk of intubation or death
						Sertraline (N = 39)	• Fluoxetine, and venlafaxine were significantly associated withlower risk of intubation or death (all *p* < 0.05)
						Fluoxetine (N = 35)	
						Citalopram (N = 27)	
						Vortioxetine (N = 3)	
						Fluvoxamine (N = 1)	

SSRI, selective serotonin reuptake inhibitor; RCT, randomized controlled trial; OR, odds ratio; CI, confidence interval; HR, hazard ratio.

## Conclusion and relevance

Long-term COVID-19 vaccine programs are on the run. However. the need for immediate effective treatment in case of life-threatening conditions underscores the importance of further investigations on the matter. The inflammatory properties of a specific class of antidepressants, SSRIs, make them potential candidates for drug repurposing in COVID-19 treatment; considering their wide range of accessibility and inexpensiveness compared to currently used anti-inflammatory agents (e.g remedsivir, bamlanivimab, casirivimab/imdevimab) (Marčec and Likić, 2021). These features have been shown to arise mostly from their ability to modulate Sig-1R activity which plays a crucial role in initiating of the IL-6 cytokine storm (Clelland et al., 2022). Also, studies suggest the protective role of Fluvoxamine, as a potent SSRI, in tissue damage induced by reactive oxygen species (Hooper, 2022).

Considering that most pulmonary complications of SARS-CoV-2 affliction originate from lung tissue damage caused by cytokine storms, this mechanism is likely to account for a part of the reduced intubation and death rates observed in several studies (Gavriatopoulou et al., 2021). Inhibition of ASM, and modulating endocytic trafficking of SARS-CoV-2, are demonstrated to be involved in anti-inflammatory pathways that are specifically associated with the COVID-19 cellular entry (Glebov, 2020). This pharmacological modulation of virus membrane entry purposes for their use as prophylactic and curative agents in the fight against the novel SARS-CoV-2. Blood clots are associated with severe clinical manifestations of COVID-19. SSRIs interact with antiplatelet medications, thus prolonging bleeding times. Cooperative administration of this class of drugs alongside anticoagulation suggests an effective first line of treatment; however, it warrants probable complications that need to be taken care (Meikle et al., 2021).

Besides all of the proposed anti-inflammatory mechanisms above, the role of negative emotions on increasing corticotropin levels, which further suppress immunity and deteriorate clinical symptoms, is another pathologic aspect of COVID-19 disease SSRIs might show anti-COVID-specific features at this level as well (Zhao et al., 2022). Further RCT studies need to be conducted to demonstrate the link between COVID-19 amelioration and negative emotion-independent effects of antidepressants like SSRIs. One paper suggested combination therapy consisting of remdesivir and fluoxetine express synergetic antiviral effect, which further requires advanced investigations (Schloer et al., 2021). Investigatory analyses have shown that SSRIs, specifically escitalopram, fluoxetine, and paroxetine, and the serotonin and norepinephrine reuptake inhibitor, vinflunine, are crucially associated with reduced risk of intubation and death (Hoertel et al., 2021); however large-scale RCTs and meta-analyses are required furthermore to approve the inclusion of SSRIs in COVID-19 treatment guidelines. At last, considering the number of humans afflicted by coronavirus over the past almost 3 years, the major problem after the severity of the disease manifestation in some sensitive cases is the emergent wave of long covid symptoms amongst the contracted population. We reported the current treatment fixed point of COVID-19 from the frame of SSRIs. New findings suggest the possible beneficial use of this type of antidepressant, especially fluvoxamine, in long covid prevention and management. This perspective inspires more detailed investigations to be conducted by researchers.
